# Cystic Adventitial Disease of Popliteal Artery with Venous Aneurysm of Popliteal Vein: Two-Year Follow-Up after Surgery

**DOI:** 10.1155/2017/4873474

**Published:** 2017-11-02

**Authors:** Koki Takizawa, Hiroshi Osawa, Atsuo Kojima, Samuel J. K. Abraham, Shigeru Hosaka

**Affiliations:** ^1^Division of Cardiovascular Surgery, Shimada General Hospital, Choshi, Japan; ^2^Department of Vascular Surgery, Tomei Atsugi Hospital, Atsugi, Japan; ^3^The Mary-Yoshio Translational Hexagon (MYTH), Nichi-In Center for Regenerative Medicine (NCRM), Chennai, India; ^4^Department of Cardiovascular Surgery, National Center of Global Health and Medicine, Shinjuku, Japan

## Abstract

We report a rare case of cystic adventitial disease of popliteal artery with venous aneurysm of popliteal vein. A 46-year-old woman had sudden-onset intermittent claudication and coldness in her right leg. The right-sided ankle-brachial pressure index (ABI) was 1.01, but peripheral arterial pulsation was decreased at knee venting position. Computed tomography revealed simple cystic lesion of the popliteal artery and stenosis of the arterial lumen in this lesion. The patient was treated by complete resection of the cystic adventitial layer of popliteal artery. A venous aneurysm of popliteal vein was revealed by intraoperative echo and was simply ligated. The patient had uneventful postoperative course and no symptoms of relevance during the two years of follow-up.

## 1. Introduction

Cystic adventitial disease (CAD) is rare vascular disorder in which a mucinous cystic formation in the adventitial layer of artery disturbs the arterial blood flow and causes intermittent claudication in young-adult patient. CAD was first reported by Atkins and Key in 1947 involving the external iliac artery [1]. CAD of popliteal artery was first described by Ejrup and Hietonn in 1954 [2]. The etiology of CAD is still controversial and several theories have been proposed. We report a case of CAD of popliteal artery with venous aneurysm of popliteal vein. The CAD was simple cystic lesion and resection of the cyst with the adventitia was successful.

## 2. Case Report

**Figure 1:**
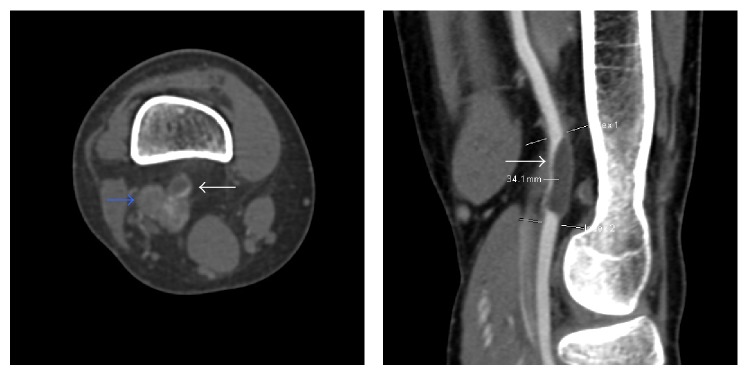
Computed tomography revealed simple cystic lesion at the popliteal artery and stenosis of the artery in this lesion. White arrow: cystic lesion of popliteal artery. Blue arrow: venous aneurysm (it was not diagnosed preoperatively).

**Figure 2:**
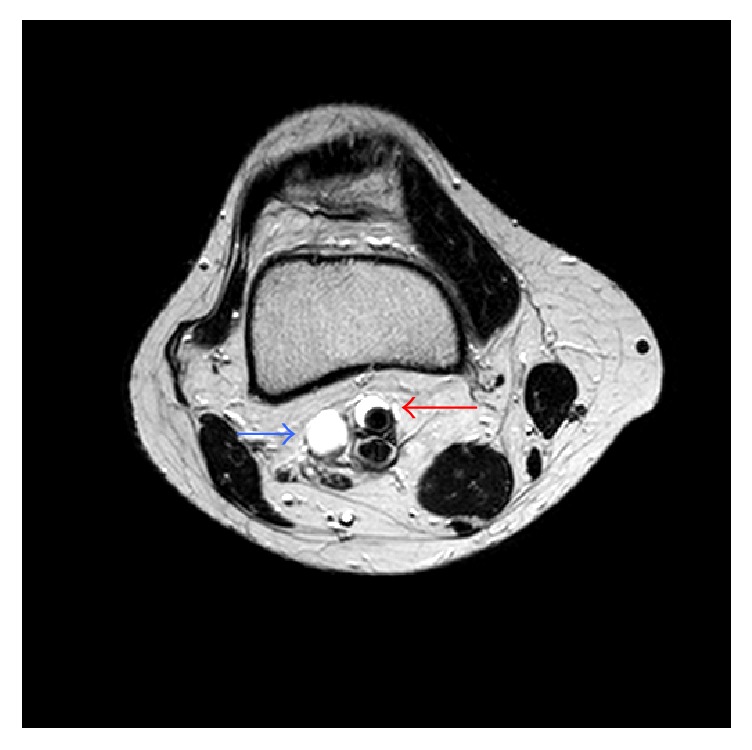
Magnetic resonance imaging (T2-weighted images) revealed cystic lesion encompassing the right popliteal artery circumferentially. Red arrow: cystic adventitial disease of popliteal artery. Blue arrow: venous aneurysm of popliteal vein (it was not diagnosed preoperatively).

A 46-year-old woman was hospitalized with sudden-onset fatigue and coldness of right leg. She also had intermittent claudication after walking 200 meters. The patient had no risk factors of vascular disease. Diminished popliteal and foot pulses, lost after knee flexion (Ishikawa sign) [3], were found on the affected limb during the clinical examination. The right ankle-brachial systolic pressure index at rest was 1.01 and the left was 1.1. Computed tomography revealed simple cystic lesion at the popliteal artery and stenosis of the artery in this lesion (Figure 1). Magnetic resonance imaging revealed cystic lesion encompassing the right popliteal artery circumferentially. This cystic lesion exhibited high signal intensity of T2-weighted images (Figure 2).

**Figure 3:**
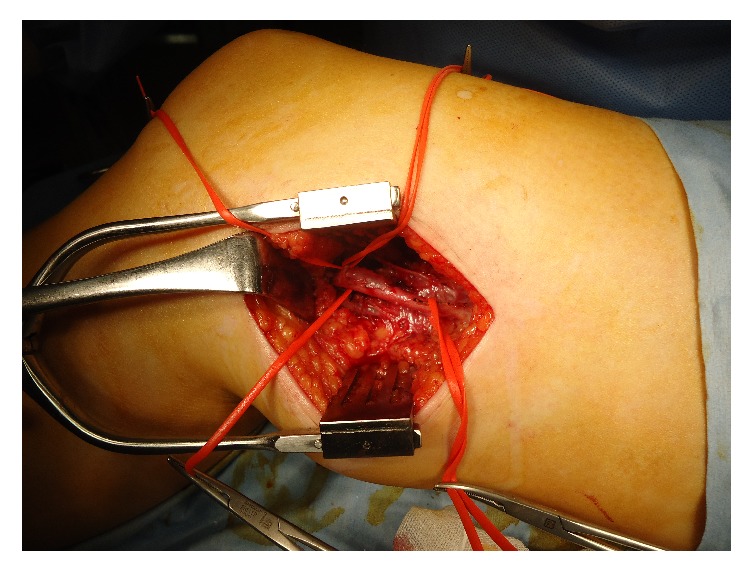
The circumferential cystic enlargement of popliteal artery.

**Figure 4:**
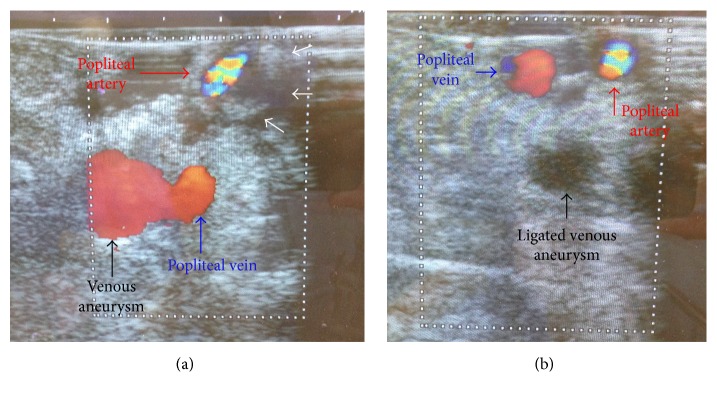
(a) Intraoperative echo findings revealed cystic adventitial lesion compressed internal lumen of popliteal artery (white arrow). (b) Intraoperative echo findings after resection of adventitial cyst revealed improved arterial compression and ligated venous aneurysm without blood flow (black arrow).

**Figure 5:**
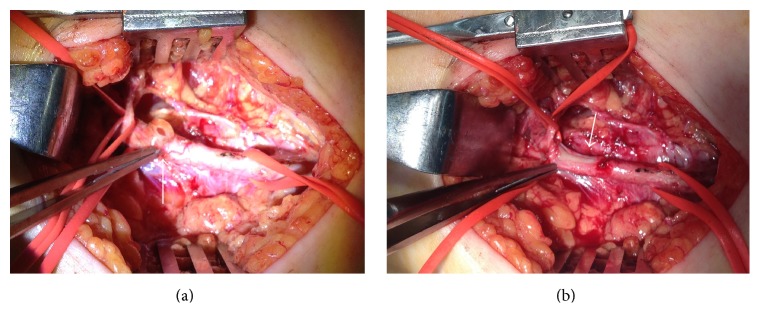
Clear viscous liquid flowed out after cutting the adventitial layer (arrow). (b) After resection of cystic adventitial lesion (arrow).

**Figure 6:**
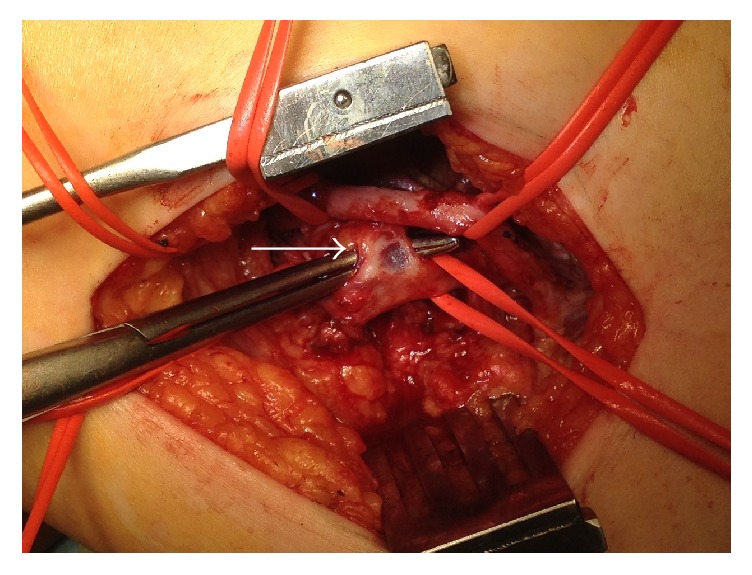
The arrow shows the neck of venous aneurysm. Venous aneurysm of popliteal vein was ligated.

The patient was diagnosed to have cystic adventitial disease and underwent surgical repair. Surgical exploration was performed through a lateral approach to expose the distal femoral artery and proximal popliteal artery. The circumferential cystic enlargement of popliteal artery was revealed (Figure 3). It was confirmed as a cystic disease by intraoperative echo (Figure 4(a)). Cutting the adventitial layer was done, when clear viscous liquid element flowed out (Figure 5(a)). Complete resection of cystic adventitial layer was made (Figure 5(b)). Arterial pulsation improved immediately (Figure 4(b)). During the procedure, a saccular venous aneurysm of popliteal vein was revealed, just lateral to the popliteal artery (Figure 4(a)), and it was ligated at the orifice (Figure 6). In fact, the venous aneurysm was revealed by preoperative computed tomography and we confirmed it during the operation (Figure 1).

**Figure 7:**
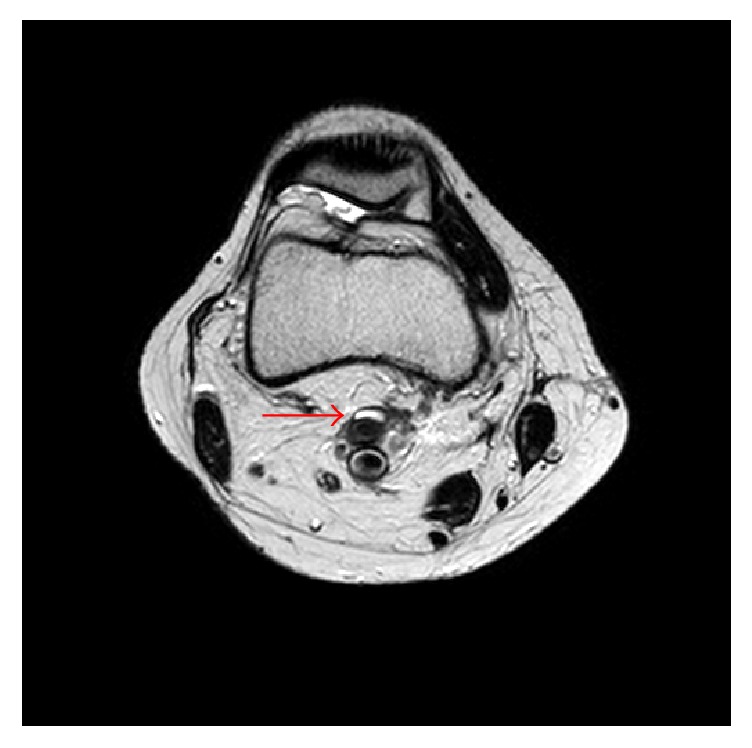
Postoperative MRI revealed 5 square mm of high intensity adventitial area (arrow) that was suspected as remaining cystic lesion.

Postoperative course was uneventful. Postoperative MRI revealed 5 square mm of high intensity adventitial area that is suspected as the remanence of the cystic lesion (Figure 7). However, two years following the surgery, patient is symptom-free with normal ankle pressure with nonchanged MRI findings.

## 3. Discussion

Cystic adventitial disease (CAD) of the popliteal artery is a rare vascular disorder in which a mucin-containing cyst develops in the adventitial layer of the artery. Several theories about pathogenesis of cystic adventitial disease have been postulated, including trauma, direct anatomic communication with the nearby joint, degeneration, and cyst formation of the adventitial layer and mucin-secretin mesenchymal cells from nearby joint [4]. Desy and Spinner described that the adventitial cyst formation begins with a capsular rent or defect that leads to the tracking of synovial fluid along an arterial articular branch [5]. Motaganahali et al. reported that 71% of patients were either active smokers or ex-smokers, nevertheless there is no relation between smoking and CAD [6].

Color-coded Doppler sonography is the most useful in revealing arterial stenosis and occlusion immediately [7]. Magnetic resonance imaging (MRI) is useful and certain to diagnose CAD. On MRI findings, the cysts are hyperintense on T2-weighted images and have low to intermediate signal intensity on T1-weighted images; it is caused by the existence of mucoid material in the cyst [8]. Multisliced direct computed tomography is very useful in evaluating arteries and planning the operating strategy.

Several treatment methods of CAD have been described following excision of the cysts and arterial segment with interposition bypass grafting, simple resection of the cyst with arterial preservation, CT or ultrasound guided percutaneous aspiration, and endovascular treatment. The treatment method should be selected according to morphology of the cystic disease. It emphasizes that the standard treatment of CAD is complete resection of the lesion including artery and graft replacement. This method is necessary for multiple cysts with severe arterial stenosis and cyst with direct communication of adjacent joint [4, 9–11]. Nevertheless, the recurrence of cystic disease in the replaced vein graft was reported [12]. Therefore strict follow-up in these patients is indispensable.

Simple resection of the cyst is suitable for simple cystic type without severe arterial stenosis and adhesion between cyst and artery. Because the recurrence is very rare in simple cystic type [8, 13].

Aspiration of cyst is not always possible because the content might be of high viscosity or the cyst is multilocular. A high recurrence rate is reported because the mucin-secretin mesenchymal cells are still present and the fluid may fill again [14].

Endovascular treatment with PTA of the popliteal artery is ineffective with unsatisfactory results. Not only is the recurrence high, but also there is a possibility of developing arterial thrombosis caused by intimal injury [4]. Therefore the endovascular treatment for cystic disease is not recommended.

In our case, cystic disease was simple and the stenosis was not severe. So we chose simple resection of the cyst and preservation of the artery. It is better than graft replacement because it will prevent the graft failure in the future.

Venous aneurysms are also considered to be a rare disease. The most common complication in venous aneurysm is deep vein thrombosis, thrombophlebitis, and recurrent pulmonary embolism [15]. Most venous aneurysms are likely to have a congenital origin [16]; however they may also be a result of degenerative changes or local inflammatory processes such as trauma and infection [17]. Surgical treatment is mandatory of popliteal vein aneurysm for the patient with thromboembolic complication [18]. The indication of surgical treatment in patients with asymptomatic venous aneurysm is controversial. However saccular aneurysm should be treated due to the high potential of future thromboembolic event [19].

About the presence of venous aneurysm nearby cystic disease in this case, there is no past case report of such a combination as far as we searched. We could not describe any comment about relation between adventitial cystic disease and venous aneurysm, but we cannot exclude that there is no relation between adventitial cystic disease and venous aneurysm.

## 4. Conclusion

Cystic adventitial disease should be suspected in middle-aged female patients who present with sudden-onset intermittent claudication of lower limb without the presence of atherosclerotic disease. Ultrasound, MRI, and CT revealed CAD of popliteal artery with simple cystic lesion and are valuable in diagnosing and deciding the strategy of operation. Simple resection of cyst was successful and the patient has no symptoms after two-year follow-up.
